# Restoring ceftolozane susceptibility: a role for diazabicyclooctane β-lactamase inhibitors?

**DOI:** 10.1128/aac.01543-24

**Published:** 2025-01-28

**Authors:** Ava J. Dorazio, Ellen G. Kline, Kevin M. Squires, Jason M. Pogue, Daria Van Tyne, Ryan K. Shields

**Affiliations:** 1 Department of Medicine, Division of Infectious Diseases, University of Pittsburgh 271847 , Pittsburgh, Pennsylvania, USA; 2 Department of Pharmacy, University of Michigan College of Pharmacy 15514 , Ann Arbor, Michigan, USA; 3 Center for Innovative Antimicrobial Therapy, University of Pittsburgh 6614 , Pittsburgh, Pennsylvania, USA; 4 Antibiotic Management Program, University of Pittsburgh Medical Center 6595 , Pittsburgh, Pennsylvania, USA; Universita degli studi di roma La Sapienza, Rome, Italy

**Keywords:** ceftolozane–tazobactam, relebactam, avibactam, durlobactam, *Pseudomonas*

## Abstract

Paired baseline and post-exposure isolates from 34 patients who developed ceftolozane–tazobactam (TOL-TAZ) resistance following treatment of multidrug-resistant (MDR) *Pseudomonas aeruginosa* infections were analyzed to determine if ceftolozane with an alternative β-lactamase inhibitor could restore susceptibility. The median baseline TOL-TAZ MIC was 2 mg/L; 88% of post-exposure isolates harbored new *ampC* mutations. Median MIC fold-increase from baseline was 32-, 24-, 16-, and 6-fold for ceftolozane–tazobactam, ceftolozane–avibactam (AVI), ceftolozane–relebactam (REL), and ceftolozane–durlobactam (DUR), respectively. Enhanced ceftolozane–durlobactam activity was evident in specific *ampC* mutations.

## INTRODUCTION

Ceftolozane–tazobactam (TOL-TAZ) is recommended as the front-line therapy for infections caused by multidrug-resistant (MDR) and difficult-to-treat resistant *Pseudomonas aeruginosa* (1, 2). Compared to ceftazidime–avibactam, treatment success rates are higher among patients treated with TOL-TAZ for MDR *P. aeruginosa* pneumonia (3); however, treatment-emergent resistance is common with both agents (3, 4). Across observational studies, the development of treatment-related TOL-TAZ resistance ranges from 3% to 50% (4–6). The primary mechanism responsible for TOL-TAZ resistance in *P. aeruginosa* involves AmpC mutations within or directly interacting with the Ω-loop region (7, 8). These mutations result in structural modifications that increase the catalytic efficiency of AmpC to TOL and other cephalosporins (9). Secondary mechanisms of resistance include mutations in AmpC regulatory genes (*ampR, ampD,* and *dacB*) that lead to hyperproduction of AmpC and mutations in penicillin-binding protein 3 (PBP3, encoded by *ftsI*) that reduce the affinity for TOL binding (8, 10).

Tazobactam does not inhibit AmpC-mediated hydrolysis of TOL and demonstrates poor binding affinity for both wild-type and mutant AmpC enzymes (9). Newer diazabicyclooctane (DBO) β-lactamase inhibitors like avibactam (AVI), durlobactam (DUR), and relebactam (REL) are potent AmpC inhibitors (11), leading us to hypothesize that combining TOL with individual DBOs may potentiate the agent’s *in vitro* activity and restore susceptibility following the emergence of TOL-TAZ resistance. To test this hypothesis, we compared minimum inhibitory concentrations (MICs) of TOL alone and in combination with TAZ, AVI, DUR, or REL among isolates collected from patients before and after they evolved TOL-TAZ resistance following treatment of MDR *P. aeruginosa* infections.

Baseline and post-exposure isolates were included from 34 patients treated for ≥48 hours with TOL-TAZ for MDR *P. aeruginosa* infection whose post-exposure isolate demonstrated resistance. Characteristics for 62% (21/34) of these patients have been previously reported (4, 8, 12, 13). Resistance was defined as a ≥4 fold MIC increase from baseline to post-exposure (4). Post-exposure isolates were collected after a median (range) treatment duration of 19.5 (4–61) days. Susceptibility testing was conducted in triplicate by broth microdilution according to Clinical and Laboratory Standards Institute (CLSI) methods (14). In brief, TOL was tested across a range of 0.25 to 256 mg/L with and without 4 mg/L of TAZ, AVI, DUR, or REL. Quality control strain *P. aeruginosa* ATCC 27853 was used throughout; results were only collected when TOL-TAZ results were within CLSI reference ranges. All isolates underwent whole-genome sequencing on the Illumina platform; genome assembly and multilocus sequence-typing were conducted as described previously (4, 8, 12). The presence or absence of β-lactamase genes was confirmed by ResFinder (15, 16). Protein sequences of baseline isolates were compared to those of PAO1; post-exposure isolates were compared to the baseline isolate from each patient for genes of interest (*ampC, ampD, ampG, ampR, dacB,* and *ftsI*). The inducible AmpC in *P. aeruginosa* is also known as *Pseudomonas-*derived cephalosporinase (PDC), which was used for consistency. Core genome single-nucleotide polymorphisms (SNPs) were compared using snippy v4.6.0 (https://github.com/tseemann/snippy), and relatedness was defined as <200 SNP difference between baseline and post-exposure isolates. All genomes are publicly available (Table S1). GraphPad Prism (version 10.2.3; Boston, MA) was used to visualize data and analyze continuous variables by the Mann–Whitney test.

Among baseline isolates, 24 unique sequence types (ST) were identified, including high-risk clones ST111 (*n* = 2) and ST308 (*n* = 1) (17); PDC-3 was the most common variant observed (*n* = 10). Post-exposure isolates varied by a median (range) of five (1–182) core-genome SNPs compared to the baseline isolate from each patient (Table S1). The median (IQR) TOL-TAZ MIC among baseline and post-exposure isolates was 2 (1–4) mg/L and 64 (28–256) mg/L, respectively. Treatment-emergent mutations in PDC were identified in 88% (30/34) of post-exposure isolates. The most common treatment-emergent mutations were G183D (*n* = 8), F147L (*n* = 7), and E247K (*n* = 5). Treatment-emergent mutations were also identified in *ampR* (*n* = 3)*, ampD* (*n* = 4)*, dacB* (*n* = 7), and *ftsI* (*n* = 6).

**Fig 1:**
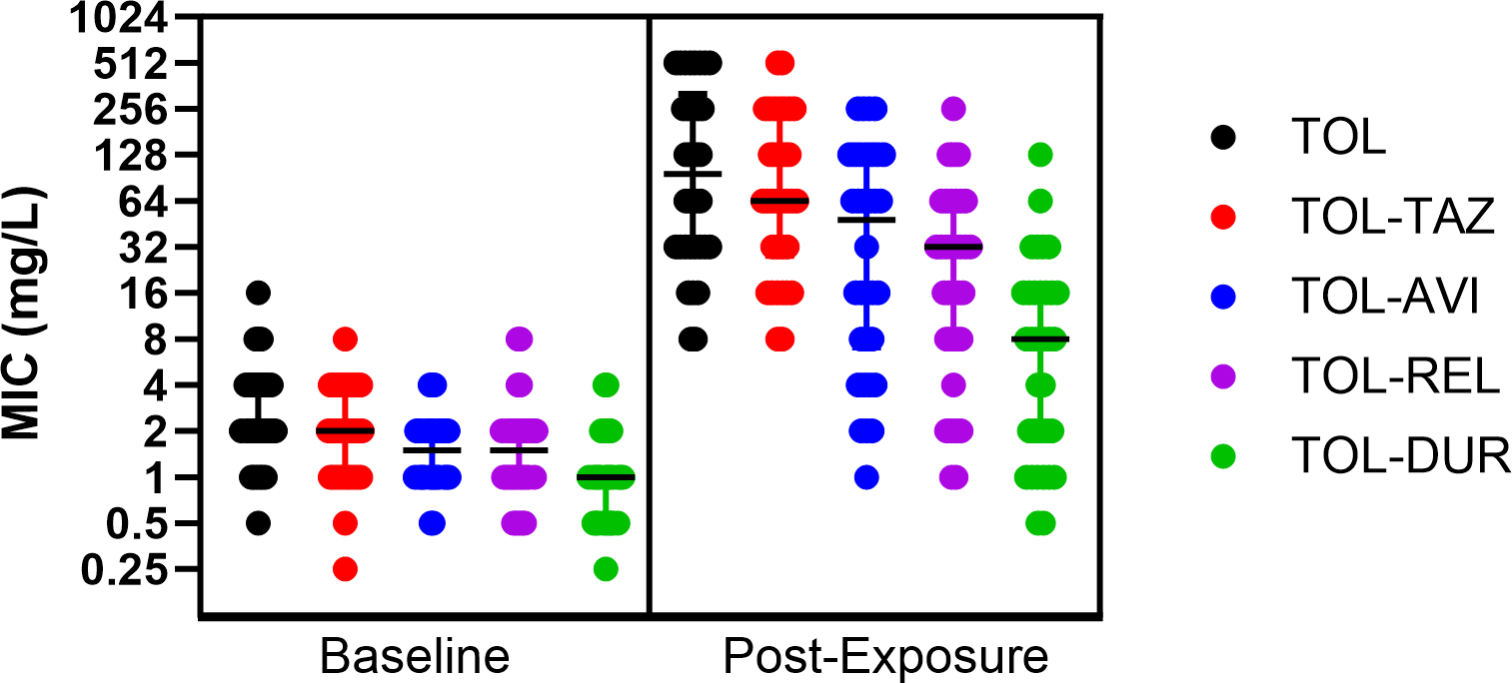
Distribution of MICs for ceftolozane-based combinations among baseline and post-exposure isolates. Note. Median MIC values are denoted by a horizontal line for each group; error bars show the interquartile range. Abbreviations: AVI = avibactam, DUR = durlobactam, MIC = minimum inhibitory concentration, REL = relebactam, TAZ = tazobactam, and TOL = ceftolozane.

The median MIC fold-increase from baseline to post-exposure isolates for TOL-TAZ was 32-fold. Corresponding MIC fold-increases for TOL-AVI, TOL-REL, and TOL-DUR were 24-, 16-, and 6-fold, respectively (Table 1). Median post-exposure MICs were lower for TOL-AVI (48 mg/L, *P* = 0.032), TOL-REL (32 mg/L, *P* = 0.0005), and TOL-DUR (8 mg/L, *P* < 0.0001) compared to TOL-TAZ (64 mg/L). Median MICs were also lower for TOL-DUR compared to TOL-AVI (*P* = 0.0003) or TOL-REL (*P* = 0.0015), resulting in a greater proportion with MICs ≤ 4 mg/L (Fig. 1). Thirty-two percent (11/34) of post-exposure isolates demonstrated a TOL-DUR MIC increase ≤2-fold from baseline.

**TABLE 1:** Distribution of ceftolozane minimum inhibitory concentrations when tested alone or with β-lactamase inhibitors among baseline and post-exposure isolates^*a*^

Drug	Baseline		Post-exposure		*P*-value^*b*^
	Median MIC (IQR) (mg/L)	Susceptible (%)	Median MIC (IQR) (mg/L)	Susceptible (%)	
TOL alone	2 (2–4)	88	96 (32–320)	0	<0.0001
TOL-TAZ	2 (1–4)	97	64 (28–256)	0	<0.0001
TOL-AVI	1.5 (1–2)	100	48 (7–128)	24	<0.0001
TOL-REL	1.5 (1–2)	94	32 (8–64)	21	<0.0001
TOL-DUR	1 (0.5–1)	100	8 (1.75–16)	44	<0.0001

^*a*^ The percent susceptible was calculated as the proportion of isolates demonstrating MIC values ≤4 mg/L, the current CLSI breakpoint for ceftolozane–tazobactam against *P. aeruginosa.* AVI = avibactam, DUR = durlobactam, MIC = minimum inhibitory concentration, REL = relebactam, TAZ = tazobactam, and TOL = ceftolozane.

^*b*^ Determined by the Mann–Whitney test comparing baseline and post-exposure MIC values.

**Fig 2:**
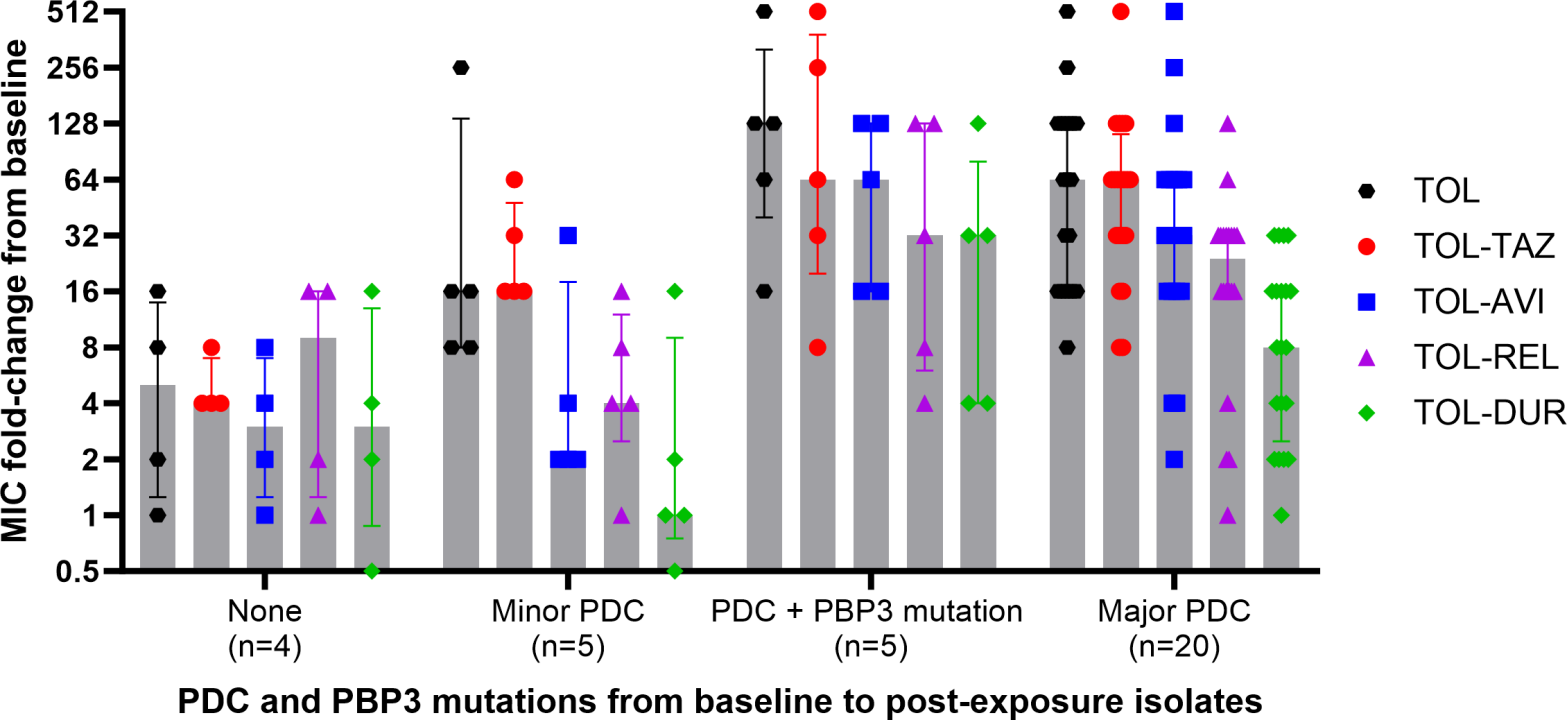
Comparison of MIC fold-changes between baseline and post-exposure isolates stratified by the underlying mechanism of ceftolozane–tazobactam resistance. Note. Median MIC values are denoted by a vertical bar for each group, and individual fold-changes for each isolate pair are shown by scatter plots. The error bars show the interquartile range. Major PDC mutations were defined as T96I, F147L, G183D, E247K, and deletions in the Ω-loop that have been previously shown to cause TOL-TAZ resistance (7, 9). Minor PDC mutations included G183R, P243S, D245del, E247G, and ins347N. Abbreviations. AVI = avibactam, DUR = durlobactam, MIC = minimum inhibitory concentration, REL = relebactam, TAZ = tazobactam, and TOL = ceftolozane.

**Fig 3:**
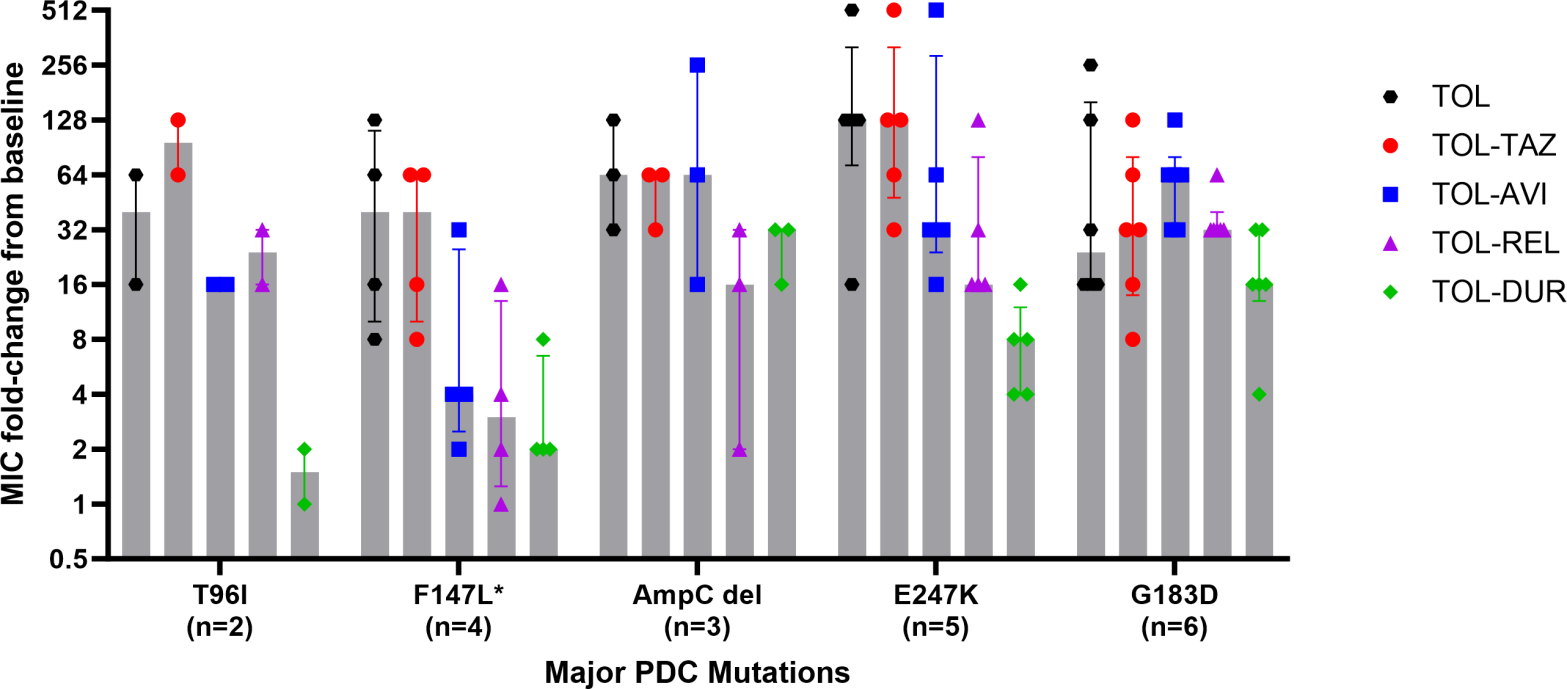
Comparison of MIC fold-changes between baseline and post-exposure isolates stratified by major PDC mutation. Note. Median MIC values are denoted by a vertical bar for each group and individual fold-changes for each isolate pair are shown by scatter plots. The error bars show the interquartile range. * Two isolates with F147L mutations harbored other major PDC mutations that were grouped with those other mutations. This included one isolate each with E247K and G183D mutations. Abbreviations. AVI = avibactam, DUR = durlobactam, MIC = minimum inhibitory concentration, REL = relebactam, TAZ = tazobactam, and TOL = ceftolozane.

Overall, 20.5% (7/34) of post-exposure isolates demonstrated ≤8-fold MIC increases compared to baseline for TOL-TAZ; 57% (4/7) of these isolates did not contain treatment-emergent PDC mutations. The remaining 27 post-exposure isolates with ≥16-fold MIC increases from baseline each harbored new PDC mutations, including amino acid substitutions (*n* = 24), deletions (*n* = 4), and/or insertions (*n* = 2). To compare MIC changes by specific PDC mutation, post-exposure isolates were grouped by likely mechanisms of resistance (Fig. 2). Mutations in PDC were categorized as major or minor mutations based on those that have been previously validated as causes of TOL-TAZ resistance (7, 9) (Table S1). Isolates with no change in PDC or those with new minor PDC mutations (*n* = 5; G183R, P243S, D245del, E247G, and ins347N) showed lower MIC-fold increases compared to isolates with major PDC mutations (*n* = 20; T96I, F147L, G183D, E247K, and deletions in Ω-loop). Isolates with additional PBP3 mutations (*n* = 5) were not significantly different than those without PBP3 mutations when major PDC mutations were also present. By comparing specific major PDC mutations (Fig. 3), we observed that the MIC fold-increase for isolates with the treatment-emergent F147L mutation was lower for all DBO combinations compared to TOL-TAZ, whereas those with T96I or E247K substitutions showed lower MIC-fold increases for TOL-DUR compared to TOL-AVI or TOL-REL. Post-exposure isolates with the G183D mutation or deletions within the Ω-loop demonstrated comparable MIC fold-increases across all agents. Additional mutations in *ampD* and/or *dacB* did not influence MIC fold-changes among isolates with major PDC mutations.

To our knowledge, this study includes the largest collection of paired baseline and post-exposure isolates collected from patients who developed TOL-TAZ resistance. These data underscore the key role PDC mutations play in conferring TOL resistance and naturally lead to the question of whether resistance can be mitigated by combining TOL with alternative β-lactamase inhibitors capable of inhibiting PDC. As such, we compared the relative MIC fold-changes of TOL-TAZ to those of TOL-AVI, TOL-REL, and TOL-DUR between baseline and post-exposure isolates. Overall, MICs increased for all TOL combinations, suggesting various levels of cross-resistance. Specifically, TOL-AVI and TOL-REL had modestly lower post-exposure MICs compared to TOL alone and showed the highest rates of cross-resistance with TOL-TAZ. These findings are consistent with the understanding that PDC mutations at residues within, or those interacting with, the Ω-loop not only lead to increased hydrolysis of TOL but also decrease the inhibitory potency of AVI (9). This is corroborated by the MIC reductions for TOL-AVI and TOL-REL in clinical isolates that were tested (Fig. 3). Median MICs were ~2-fold lower for TOL-REL compared to TOL-AVI against isolates with major PDC mutations. By comparison, the MIC fold-changes for TOL-DUR were specific to the mutation identified but lower than those for TOL-TAZ, TOL-AVI, or TOL-REL. For example, the median MIC fold-increase from baseline was similar for TOL-DUR (16-fold) and TOL-TAZ (32-fold) against isolates with G183D mutations. By comparison, isolates with T96I or E247K mutations showed significantly lower MIC fold-increases for TOL-DUR (4-fold) compared to TOL-TAZ (128-fold). These data support the hypothesis that specific structural changes in PDC impact the inhibitory activity of DUR less than AVI or REL. Indeed, DUR demonstrates a greater inhibitory potency than AVI for many serine β-lactamases, including PDC (18). It is also possible that the inhibitory activity of DUR was potentiated by inhibition of PBP2 (19). While DUR demonstrates less binding affinity for PBP2 in *P. aeruginosa* compared to PBP2 in *Escherichia coli* or *Acinetobacter baumannii*, important morphologic changes occur upon exposure that may impact *in vitro* growth (19).

It has now been established that major PDC mutations (T96I, F147L, G183D, ΔG229-E247, and E247K) result in structural modifications that widen the binding pocket and increase TOL hydrolysis (7–9, 20); however, several other secondary mechanisms of TOL-TAZ resistance have been reported (8). In fact, 26% (9/34) of post-exposure isolates demonstrating nonsusceptibility to TOL-TAZ (median post-exposure TOL-TAZ MIC = 16 mg/L) did not harbor major mutations in PDC or PBP3. Among those nine isolates, 33%, 44%, and 78% demonstrated TOL-REL, TOL-AVI, and TOL-DUR MICs ≤ 4 mg/L, respectively, the current TOL-TAZ susceptibility breakpoint. Corresponding median MICs were 8, 8, and 2 mg/L, respectively. Given the relative exposures of TOL achieved in patients (21), studies are needed to determine if isolates with minor PDC mutations or those with only mutations in PDC regulatory genes (*ampD, ampR,* and *dacB*) could be treated with TOL plus a DBO inhibitor. Currently, TOL-TAZ is only commercially available as a fixed combination, and rapid molecular diagnostic tests do not differentiate PDC resistance mechanisms; however, designing new β-lactam/β-lactamase inhibitor (BL/BLI) combinations to overcome MDR *P. aeruginosa* resistance mechanisms is a key priority. This is especially true given that more than 20% of patients with MDR *P. aeruginosa* infections treated with TOL-TAZ or ceftazidime–avibactam develop resistance within 90 days (3). Because cross-resistance occurs between TOL-TAZ, ceftazidime–avibactam, and even cefiderocol (8, 12), identifying new BL/BLI combinations like TOL-DUR may prove valuable in combatting MDR *P. aeruginosa* infections with limited treatment options.

## SUPPLEMENTAL MATERIAL

Table S1 aac.01543-24-s0001.xlsx Molecular characteristics and antibiotic susceptibility results for MDR *P. aeruginosa* isolates.

ASM does not own the copyrights to Supplemental Material that may be linked to, or accessed through, an article. The authors have granted ASM a non-exclusive, world-wide license to publish the Supplemental Material files. Please contact the corresponding author directly for reuse.
